# Electrochemical Oxidation and Determination of Antiviral Drug Acyclovir by Modified Carbon Paste Electrode With Magnetic CdO Nanoparticles

**DOI:** 10.3389/fchem.2020.00689

**Published:** 2020-09-10

**Authors:** Ebrahim Naghian, Elnaz Marzi Khosrowshahi, Esmail Sohouli, Hamid Reza Pazoki-Toroudi, Ali Sobhani-Nasab, Mehdi Rahimi-Nasrabadi, Farhad Ahmadi

**Affiliations:** ^1^Chemical Injuries Research Center, Systems Biology and Poisonings Institute, Baqiyatallah University of Medical Sciences, Tehran, Iran; ^2^Faculty of Pharmacy, Baqiyatallah University of Medical Sciences, Tehran, Iran; ^3^Department of Chemistry, South Tehran Branch Islamic Azad University, Tehran, Iran; ^4^Faculty of Pharmacy, Tabriz University of Medical Sciences, Tabriz, Iran; ^5^Young Researchers and Elites Club, Science and Research Branch, Islamic Azad University, Tehran, Iran; ^6^Physiology Research Center, Iran University of Medical Sciences, Tehran, Iran; ^7^Social Determinants of Health (SDH) Research Center, Kashan University of Medical Sciences, Kashan, Iran; ^8^Core Research Lab, Kashan University of Medical Sciences, Kashan, Iran; ^9^Department of Medicinal Chemistry, School of Pharmacy-International Campus, Iran University of Medical Sciences, Tehran, Iran

**Keywords:** acyclovir, antiviral drug, carbon paste electrode, electrochemical sensor, magnetic cadmium oxide, nanoparticle

## Abstract

With the development of nanomaterials in electrochemical sensors, the use of nanostructures to modify the electrode surface has been shown to improve the kinetics of the electron transfer process. In this study, a sensor was developed for the electrochemical determination of Acyclovir (ACV) based on the modified carbon paste electrode (CPE) by CdO/Fe_3_O_4_. The magnetic CdO nanoparticles characterization was studied by energy-dispersive X-ray spectroscopy (EDS) and X-ray diffraction (XRD). To study of the modified CPE surface morphology, scanning electron microscopy (SEM) was used. At the optimal conditions, a noteworthy enhancement in the electrochemical behavior of ACV was observed at the surface of the modified CPE compared to the unmodified CPE. A detection limit of 300 nM and a linear range of 1–100 μM were obtained for the quantitative monitoring of ACV at the modified CPE surface using differential pulse voltammetry (DPV) in phosphate buffer. The RSD% (relative standard deviation) of the electrode response was <4.3% indicating the development of a high precision method. Also, satisfactory results were obtained in the determination of ACV with the modified electrode in tablet, blood serum, and urine samples with a satisfactory relative recovery (RR%) in the range of 94.0–104.4%.

## Introduction

Herpes simplex is a type of skin infection caused by the herpes hominies virus (Braun-Falco et al., 1991) and is commonly known as herpes blister. There are two different types of herpes simplex virus types 1 and 2 of the herpes virus family that infect humans. The immune system removes the virus from the skin, but the virus hides itself in the nerves and may reappear in the future. 2-amino-9-[(2-hydroxyethoxy) methyl]-6,9-dihydro-3H-purin-6-one or acyclovir (ACV) is one of the effective drugs for reduces of this virus (Piret and Boivin, 2017). ACV is an antiviral drug with significant activity. It plays a significant role in the treatment of the virus illnesses and is very distinctive in helping the body to fight infection effectively and provide the necessary immunity. ACV does not completely cure herpes disease, but it certainly works to reduce the symptoms and spread herpes infection (Kłysik et al., 2020; Luyt et al., 2020). ACV is widely used in the therapy of herpes zoster infections, herpes simplex, herpetic encephalitis, primary genital herpes, and varicella-zoster virus infections in immunosuppressed patients. It is also helpful in inhibiting HSV infections in renal allograft receptors (Tenser and Tenser, 2019) and its anti-hepatitis B virus activity has been demonstrated (Huang et al., 2011). ACV may lead to neurotoxicity (coma, hallucinations, lethargy, tremors, and seizures) and nephrotoxicity (crystallization of ACV within renal tubules, transient, and enhancement of serum creatinine) (Fleischer and Johnson, 2010). As ACV is structurally similar to endogenous substances and because of its high solubility in water, it is very difficult to isolate and measure it in biological fluids. Therefore, its analysis in human serum is complicated and requires high selective analytical methods. With notice of these facts, the quantitative monitoring of ACV in biological fluids and other real samples appears very important. Several analytical methods have been developed for the analysis of ACV including near-infrared spectroscopy (Yu and Xiang, 2008), fluorimetric and photometric methods (Macka et al., 1993), radioimmunoassay (RIA) (Tadepalli and Quinn, 1996), and LC (liquid chromatography) (Tzanavaras and Themelis, 2007; Mulabagal et al., 2020). The mentioned methods are commonly needed expensive equipment, more time and tedious processes. For example, LC methods need to optimize the chromatographic conditions and samples preparation or RIA method hands the radioactive wastes. Although, electrochemical methods including differential pulse voltammetry (DPV), cyclic voltammetry (CV), square wave voltammetry, amperometry, electro-chemiluminescence, and polarography (Wang et al., 2006, 2013; Heli et al., 2010; Shetti et al., 2012, 2017; Karim-Nezhad et al., 2018) provide advantages such as simplicity, high sensitivity, low cost, fast response, and more environmentally friendly techniques (Adib et al., 2016; Rahimi-Nasrabadi et al., 2017; Amani et al., 2018a,b; Khoshroo et al., 2018, 2019; Naghian et al., 2020; Sanatkar et al., 2020; Sohouli et al., 2020a,b). Metal oxide nanoparticles have been extensively developed in the past decades. Among the metal oxide nanoparticles, cadmium oxide nanoparticles (CdONPs) are attractive because of wide non-toxicity, high isoelectric point and large surface area. So, CdNPs is a promising candidate for support material in the construction of the biosensors (Jing and Bowser, 2011; Fouladgar, 2018; Malekmohammadi et al., 2018; Kumar H.C. et al., 2019; Maduraiveeran et al., 2019; Mitra et al., 2019). metal nanoparticles modified electrodes show rapid redox activity toward the compounds with slow electron kinetics at bare electrodes, resolve overlapped peaks of analytes with close oxidation potentials and reveal good peak to peak separations (Baig et al., 2019; Sajid and Baig, 2019; Sinha et al., 2019). Metal ferrite or magnetic compounds have long been considered owing to its unique properties such as chemical and thermal stability, lower toxicity relative to other metals and cost-effectiveness. The most special and important property of metal ferrite for electrochemical applications is its good conductivity which originates from charge hopping of carriers between cations occupying the octahedral site (Beitollai et al., 2019; Khorshed et al., 2019; Kumar S. et al., 2019; Pastucha et al., 2019).

In this paper, a novel magnetic cadmium oxide CdO/Fe_3_O_4_ modifier was used to modify a carbon paste electrode (CPE). The electrocatalytic activity of the modified CPE was investigated for the detection and analysis of ACV using DPV. The interaction between cadmium ion and ACV enhances the accumulation of the drug on the surface of the modified electrode and increasing the sensitivity of the measurement. After optimization of the experimental parameters, the modified electrode was used for analyses of the low level of ACV in real samples including biological fluid (plasma and urine) and tablet.

## Experimental

### Instrumentation

Voltammetric measurements were performed using a three-electrode system consisting of the Ag/AgCl electrode as the reference electrode (Azar Electrode Co., Iran), the platinum wire as the auxiliary electrode (Azar Electrode Co., Iran) and a modified CPE as the working electrode. A μ-Autolab type III/FRA2 potentiostat/galvanostat with NOVA software was used for impedance measurements. To determine the structure and morphology of synthesized magnetic nanoparticles, FESEM images were obtained the SIGMA VP field emission scanning electron microscope coupled with EDS analysis and FEI NOVA NanoSEM450 instrument. X-ray diffraction (XRD) patterns were recorded by a Philips-X'pertpro, diffractometer containing Ni-filtered Cu K*a* radiation. The Metrohm pH meter (Model 691) was used to adjust the solutions' pH. The voltammograms were recorded via the PSTrace software and all data analysis were performed using Excel software.

### Chemicals and Reagents

All chemicals were prepared in high purity analytical grade from Merck Company (Germany). ACV standard powder was obtained from the Rouz Darou Company. The Stock solutions of ACV were prepared by dissolving the appropriate amounts in a suitable volume of water before analysis. Other standard solutions were obtained by diluting the stock solutions with phosphate buffer solution.

### Synthesis of Fe_3_O_4_ Nanoparticles

The Fe_3_O_4_ magnetic nanoparticles were synthesis by a reaction between 0.02 mol of iron (II) chloride and 0.03 mol of iron (III) chloride. These salts were dissolved in a 40 mL of degassed HCl (0.4 M) solution and then 375 mL of degassed ammonia solution (25%) was added to the above solution during 45 min drop by drop under sonication. After that, the black precipitate (Fe_3_O_4_ nanoparticles) was formed. Finally, the separated products were washed with ethanol and deionized water and dried in an oven at 70°C (Enayat, 2018).

### Synthesis of CdO/Fe_3_O_4_ Nanoparticles

The synthesized Fe_3_O_4_ nanoparticles were dispersed in 50 mL of deionized water. The dispersion was stirred softly for 10 min to become homogenous. One millimole of cadmium nitrate warm solution (70°C) and Fe_3_O_4_ dispersion were added to 50 mL of NaOH with a concentration of 2.5 mol L^−1^ under stirring conditions for 15 min. Finally, the CdO/Fe_3_O_4_ precipitation was rinsed with distilled water and after drying, it was calcined at 450°C for 70 min (Vosoughifar, 2018).

### Preparation of Modified Carbon Paste Electrode With Magnetic Nanoparticles

0.14 g of graphite, 0.01 g of magnetic nanoparticles and some oil were mixed to achieve a uniform paste. The resulting paste was then put into a plastic tube (id = 2 mm). The electrical transmission was made using a copper wire. The surface of the electrode was polished on a paper sheet to be perfectly uniform. The unmodified CPE was obtained from a mixture of 0.15 g of graphite and oil with no modifier similar to the mentioned procedure.

### Tablet, Urine, and Plasma Samples Pretreatment

To prepare tablet sample (ROUZ DAROU Co.), 10 tablets (each containing 200 mg of ACV) were weighed and uniformly powdered. The 225 mg of the obtained powder was dissolved in 100 mL of 0.1 M phosphate buffer (pH = 4). The resulting solution was diluted 500-folds and stirred for 10 min to dissolve completely and finally it was filtered before use. Urine and plasma samples were stored in the refrigerator after collection. Then 5 ml of the solutions were centrifuged for 5 min at 5,000 rpm (to remove suspended particles. These particles sometimes contaminate the surface by absorbing on the electrode and reduce its efficiency). The dilution with phosphate buffer (pH = 4) was done at a ratio of 0.1 to reduce the matrix effect. The ACV content of samples was measured using the standard addition method.

## Results and Discussion

### EDS and XRD Analysis of As-Synthesized Magnetic CdO

**Figure 1:**
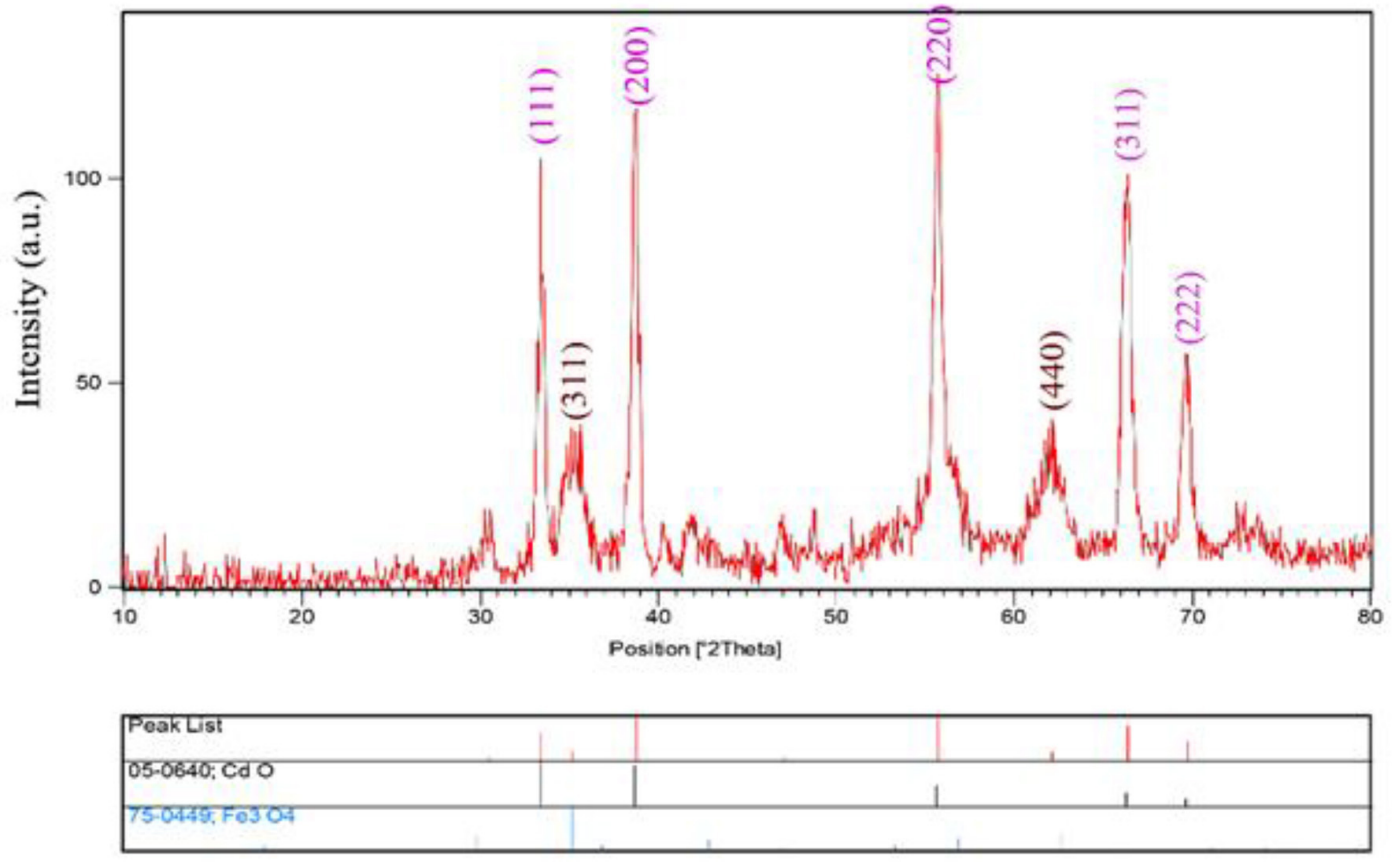
The XRD pattern of CdO/Fe_3_O_4_.

**Figure 2:**
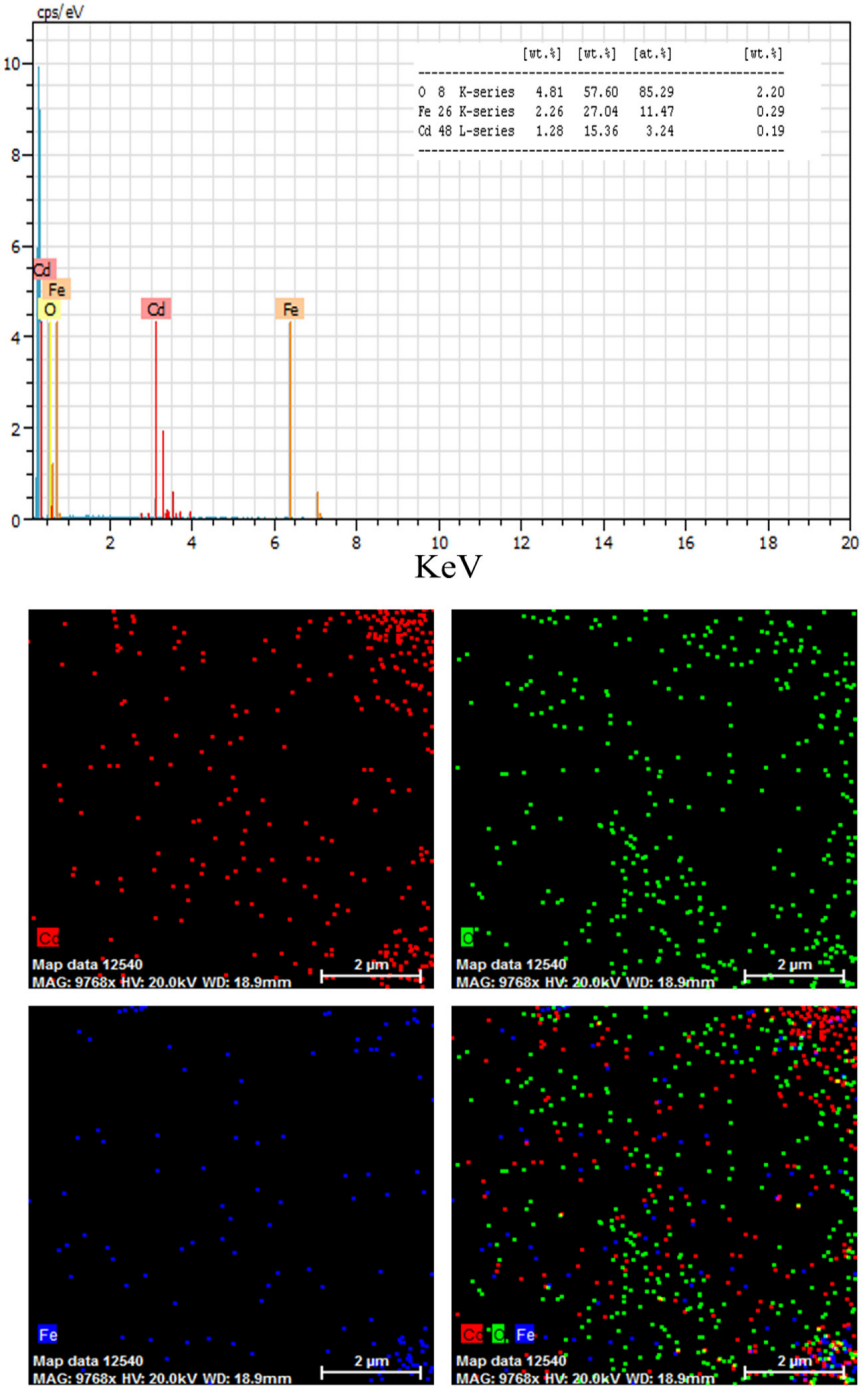
The results of EDS analysis for CdO/Fe_3_O_4_ nanoparticles.

The XRD patterns of CdO/Fe_3_O_4_ nanostructures have been presented in Figure 1. As our results advice, CdO/Fe_3_O_4_ nanostructure has high purity and two phases. The first phase is the Fe_3_O_4_ sample shows a series of diffraction peaks at the position of 35.45° [(311) line], 43.08° [(400) line], 57.16° [(511) line], and 62.72° [(440) line] that is in good agreement with the standard JCPDS file of Fe_3_O_4_ Cubic phase (space group Fd-3m, JCPDS No. 75-0449) and CdO shows a series of diffraction peaks at the position of 33.35° [(111) line], 38.78° [(200) line], 55.52° [(220) line], 65.92° [(311) line], and 69.33° [(222) line] which is in good agreement with the standard JCPDS with the crystal structure of tetragonal (JCPDS 75-0592) with a space group of Fd-3m, respectively (Vosoughifar, 2018). Crystalline sizes of nanoparticles are calculated from Scherer equation:

$$\begin{array}{l} \text{Dc}=\text{K}\lambda /\beta \text{Cos}\theta \end{array}$$

where K is the shape factor, λ is the X-ray wavelength (Cu Kα radiation, equals to 0.154 nm), β is the width of the observed diffraction peak at its half maximum intensity, which takes a value of about 0.9 and θ is the angle between the incident beam and the sample surface. The CdO/Fe_3_O_4_ nanoparticles sizes were about 23 nm. The EDS analysis approves the presence of Cd, Fe and oxygen elements with no impurity (Figure 2).

### Optimization of the Percentage of Magnetic Nanoparticles

To achieve the high-performance CPE, a suitable ratio between oil and graphite is needed (9–10%). Higher and lower percentages are not suitable because the high amounts of oil obtain a flowing liquid and increase the surface hydrophobicity. In lower oil percentages, the electrode absorbs water and falls apart. However, the percentage of nanoparticles should be optimized due to the effect on sensor sensitivity and signal amplification. For this purpose, CPE with different weight ratios of magnetic CdO: graphite (3, 5, 7, and 10%) were prepared and its efficiency for measuring 30 μA of ACV was evaluated. The results showed that from 3 to 10 incremental trends are observed in the signal, but after 7% this trend continues with a lower slope, so for optimal consumption of nanoparticles, this value was used as the optimal percentage in the structure of the electrode.

### Working Electrode Surface Area and Microscopic Imaging

**Figure 3:**
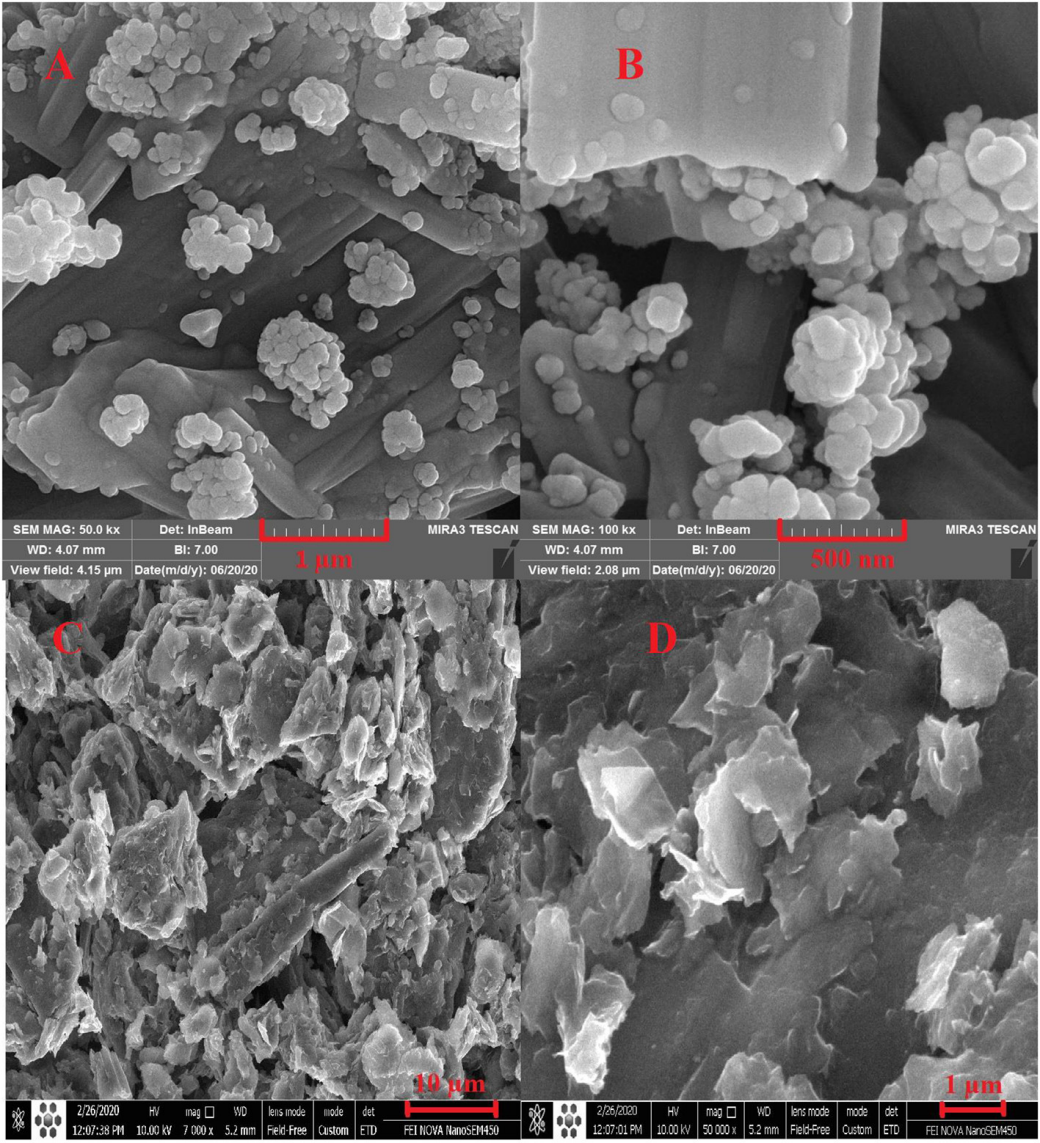
The SEM images of magnetic CdO nanoparticles **(A,B)** and carbon paste with magnetic CdO **(C,D)**.

The SEM technique was used to illustrate the morphology of magnetic nanoparticles after being embedded in the carbon paste matrix. Figure 3 shows that the CPE plate morphology becomes cavernous by adding nanoparticles and the surface area increases significantly. The enhancement of magnetic nanoparticles provides a more porous structure for the CPE and increases the active surface area of the electrode in contact with the electrolyte and the electroactive species.

The effective surface area of the electrode (*A*, cm^2^) was calculated from obtained CV data of 5 mM K_3_[Fe(CN)_6_] with a diffusion coefficient (*D*, Cm^2^ s^−1^) of 7.5 × 10^−6^. It was calculated from the Randles-Sevcik equation (Bard and Faulkner, 2001):

$$\begin{array}{l} I_{pa}=2.69\times 10^{5}n^{3/2}AC_{0}D^{1/2}v^{1/2} \end{array}$$

where *n* is the number of transferred electrons in the oxidation and reduction process of ferrocyanide, *C* is the concentration of ferrocyanide (5 × 10^−9^ mol cm^−3^) and ν is the scan rate. The value of CdO/Fe_3_O_4_/CPE surface area (0.31 cm^2^) is greater than the unmodified CPE (0.14 cm^2^) which can be lead to exist of more electrochemical reaction sites.

### Electro-Oxidation Behavior of the Redox Probe and ACV on the Modified CPE

**Figure 4:**
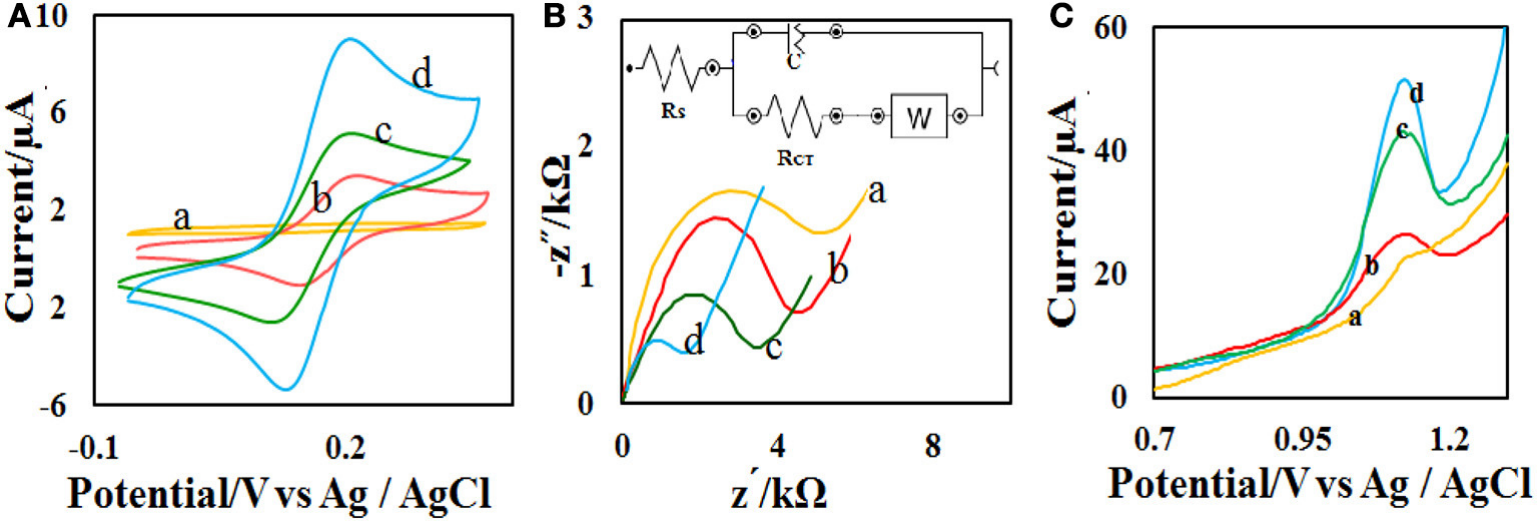
**(A)** CVs and **(B)** Nyquist plots of the different modified electrodes in 0.1 M PB containing 5.0 mM [Fe(CN)_6_]^−3/−4^ at (a) bare, (b) CdO NP/CPE, (c) Fe_3_O_4_ NP/CPE, and (d) CdO/Fe_3_O_4_ NP/CPE in the scan rate of 50 mV s^−1^ **(C)** The recorded DPVs of the (a) bare, (b) CdO NP/CPE, (c) Fe_3_O_4_ NP/CPE, and (d) CdO/Fe_3_O_4_ NP/CPE in presence of 50 μM ACV.

The CV technique was employed to evaluate the performance of the prepared electrodes. For this purpose, electrochemical measurements were done in the mixture of Fe^2+^/Fe^3+^ aqueous solution (5 mM, molar ratio 1:1) containing 0.1 M potassium chloride in the potential range of −0.3 to 0.7 V (Figure 4A). At the CPE surface, the oxidation and reduction peaks of Fe^2+^/Fe^3+^ are very weak. However, when the electrode was modified with CdO/Fe_3_O_4_ an oxidation-reduction peak for Fe^2+^/Fe^3+^ solution showed a difference between the peaks of 80 mV indicating that this electrode has better capability rather than the unmodified electrode.

EIS (electrochemical impedance spectroscopy) was used to measure the surface resistance of different electrodes. The purpose of this section is to examine changes in the rate of electron transfer at different electrode surfaces. Figure 4B presents the Nyquist plots in the presence of Fe^2+^/Fe^3+^ aqueous solution (5 mM) and 0.1 M potassium chloride solution. Semicircular diameter indicates electron transfer resistance (*R*_ct_) of the redox probe reaction. The *R*_ct_ parameter in this technique indicates the reaction kinetics at the electrode surfaces. The value of *R*_ct_ for CPE (5 kΩ) bigger than the modified CPE (1.2 Ω). This behavior is due to the accelerate of the probe redox electron transfer at the CdO/Fe_3_O_4_/CPE surface.

The electro-oxidation behavior of the analyte on the CdO/Fe_3_O_4_/CPE surface was examined by the DPV method (Figure 4C). Comparison of the recorded DPVs of the bare, CdO/CPE, Fe_3_O_4_/CPE, and CdO/Fe_3_O_4_/CPE in presence of 50 μM ACV shows that the oxidation of ACV on the CdO/Fe_3_O_4_/CPE surface has a higher current and lower potential.

### Effect of Electrolyte pH and Reaction Mechanism

**Figure 5:**
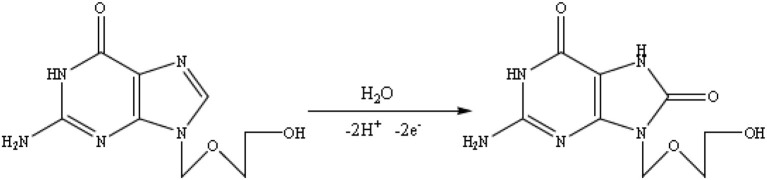
The ACV oxidation mechanism.

**Figure 6:**
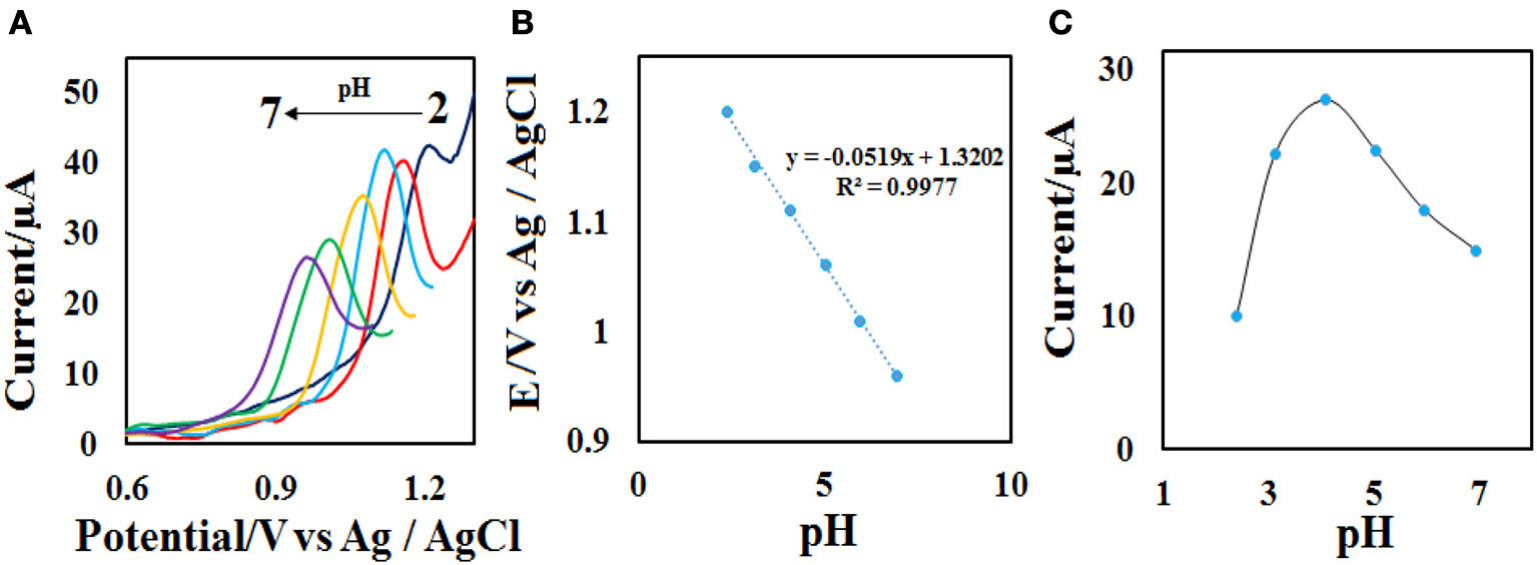
**(A)** DPVs of the CdO/Fe_3_O_4_/CPE at various pH values (2, 3, 4, 5, 6, and 7). **(B)** Plots of the *E*_*p*_ vs. pH value and **(C)** *I*_*p*_ vs. pH value in the presence of 50 μM ACV.

The effect of solution pH on the ACV oxidation peak in the pH ranges from 2 to 7 was investigated in the presence of phosphate buffer as a supporting electrolyte. The oxidation potential of ACV changed with increasing pH. This indicates that the proton is involved in oxidative reactions. The oxidation potential of ACV is shifted to negative values by changing pH. According to the structure of ACV, the electrochemical oxidation process of ACV participates is equal protons and electrons (Figure 5). This mechanism is also confirmed by electrochemical methods presented in the previous work (Shetti et al., 2017). So the pH is important for the determination of ACV and its maximum oxidation current at developed sensor was obtained in pH = 4 that used for further studies (Figure 6).

### Effect of Scan Rate

**Figure 7:**
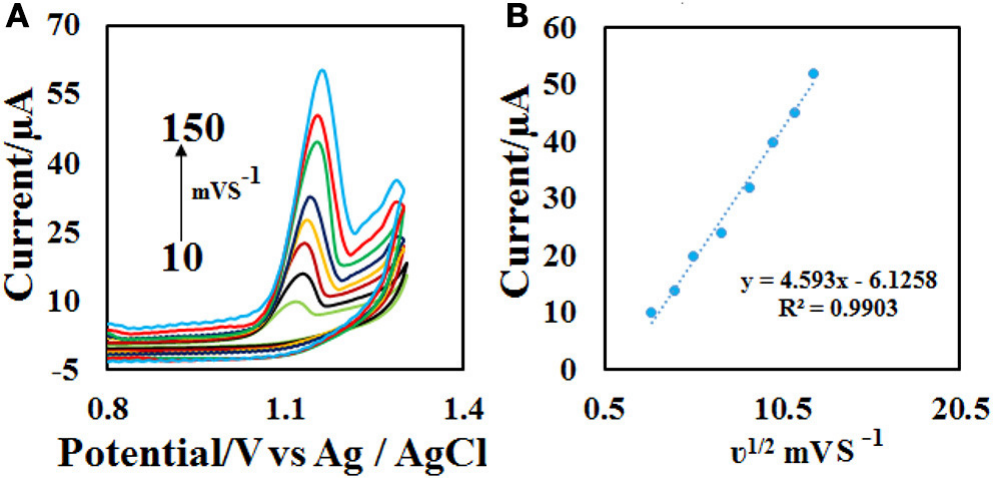
**(A)** CVs on the CdO/Fe_3_O_4_/CPE in 0.1 M phosphate buffer (pH = 4.0) containing 50 μM ACV solution at different scan rates (10–150 mVs^−1^) and **(B)** the plot of **(B)** *I*_*p*_ vs. υ^1/2^.

The effect of scan rate on the electrochemical properties of the CdO/Fe_3_O_4_/CPE was investigated by the recording of the related voltammogram at different scan rates from 10.0 to 150.0 mV s^−1^ in the 0.1 M phosphate buffer containing 50 μM ACV. As seen in Figure 7A, with the increase of scan rates the oxidation peak current increases and the E_p_ value, slightly increases. The relationship between the oxidation peak currents (*I*_p_) vs. the square root of the scan rate (υ^1/2^) was obtained to be linear in the range of 10.0–150.0 mV s^−1^ under the corresponding equations including *I*_pa_= 4.593 υ^1/2^ −6.1258 (Figure 7B). These results emphasize a diffusion-controlled oxidation process at the CdO/Fe_3_O_4_/CPE surface.

### Analytical Variation and Comparison of the Proposed Method With Other Electrosensors

**Figure 8:**
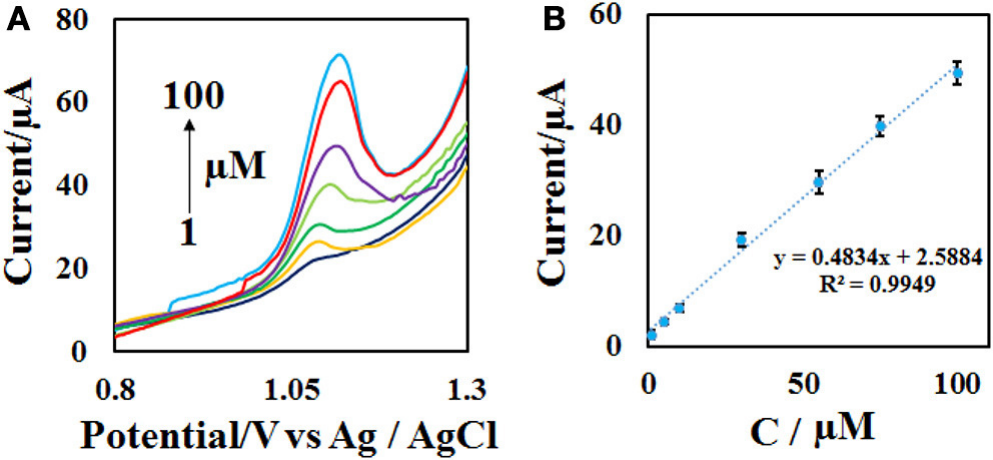
**(A)** DPVs of the CdO/Fe_3_O_4_/CPE in the presence of 1–100 μM ACV in 0.1 M phosphate buffer (pH = 4) and the scan rate of 50 mV s^−1^ and **(B)** the calibration curve.

To achieve the working range of the developed sensor, the DPV of different concentrations of ACV (Figure 8A) were obtained at the optimal condition (0.1 M phosphate buffer with pH = 4.0 and the scan rate = 50 mV s^−1^) with 3 replicates. The plotting of current vs. concentration shows that the linear range of the method is in the range of 1–100 μM with a detection limit of 0.3 μM (Figure 8B). Other analytical performance parameters including repeatability and stability were investigated according to follow.

To investigate the repeatability of the modified electrode response, the ACV related voltammograms were plotted 5 times at three different concentrations and the RSD (relative standard deviation) of the electrode response was calculated. RSD values of the electrode response were <4.3%. The stability of the modified CPE was checked with the recording of ACV voltammograms once a week consecutively for 8 weeks. The results indicate that the desired CdO/Fe_3_O_4_/CPE surface has long-term stability and in the eighth week, only a 5% signal decrease was observed compared with the first week.

In a comparison of the developed method with the other proposed methods for the measurement of ACV (Table 1) appears some advantages of this method. The used electrode is economical in comparing of glassy carbon electrode (GCE) or dispersed nanoparticles modifiers. Other analysis parameters such as the detection limit and the linear range are comparable and in some cases better than the presented methods.

**Table 1:** Comparison of the method with other electrochemical methods.

**Modifier/electrode**	**Linear range (μM)**	**LOD (μM)**	**References**
GCE/MWCNT/ZnO	0.009–1	0.006	Karim-Nezhad et al., 2018
CPE/Nnao clay	0.05–1	0.0002	Shetti et al., 2017
GCE/MWCNT-DHP^a^	0.79–130	0.16	Wang et al., 2006
GCE/C60	0.8–6	0.15	Shetti et al., 2012
CPE/PVP^b^	0.01–75	0.03	Wang et al., 2013
CPE/CuNPs	27–521	2.6	Heli et al., 2010
CdO/Fe_3_O_4_/CPE	1–100	0.3	This work

a *Dihexadecyl hydrogen phosphate*.

b *Polyvinylpyrrolidone*.

### Interfering Effect

**Figure 9:**
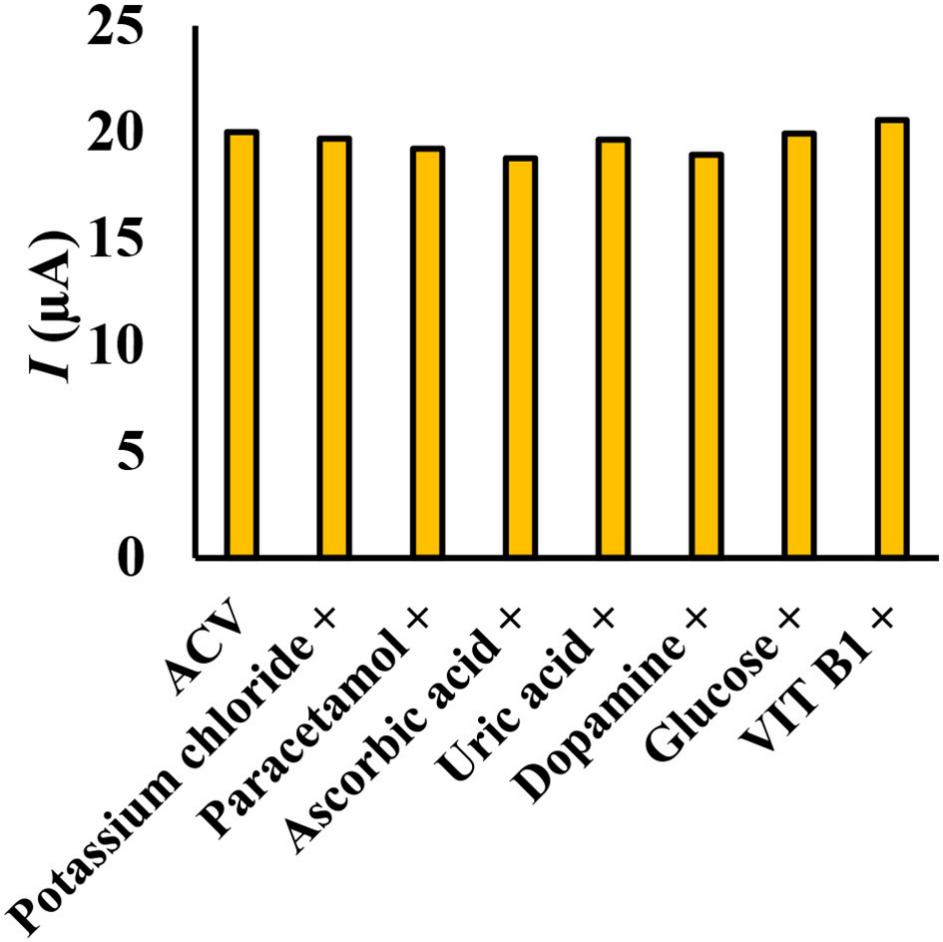
Simultaneous determination of ACV in the presence of some interfering species (ratio >100), the experimental conditions: 0.1 M phosphate buffer, pH = 4, scan rate = 50 mVs^−1^, ACV concentration = 30 μM.

To measure ACV in different matrices, it is necessary to investigate the effect of species that can oxidize or reduce on the electrode surface. The effect of different substances on the potential interaction of ACV determination at optimum conditions was studied. Interfering substances were selected from the group of substances present in biological and pharmaceutical fluids. The nuisance threshold was determined as the maximum species with an error of <5% in the determination of ACV. The results (Figure 9) showed that the presence of glucose, ascorbic acid, dopamine, uric acid, paracetamol, acetaminophen, vitamin B1, and potassium chloride did not affect the sensor selectivity. It should be noted that these species do not have any overlapped peak in the same peak potential of ACV.

### Real Sample Analysis

To investigate the application of the modified electrode in the electrochemical determination of ACV, tablet, urine and plasma samples were selected. Table 2 shows the results of this analysis. The results confirm the high performance of the modified electrode for measuring ACV in real samples. Each sample was spiked with three levels of ACV in the calibration range and the amount of ACV was obtained from the standard addition method. To achieve the relative recovery (RR) of the method, the ratio of ACV analytical signal in phosphate buffer and real samples was calculated. The recovery results (94.0–104.4%) indicate good accuracy for the presented method in real samples.

**Table 2:** Determination of ACV content in real samples with relative recovery.

**Sample**	**Added (μM)**	**Found (μM)**	**RR (%)**	**RSD (%), *n* = 5**
Tablet (200 mg)	0	20.2	100.1	2.3
	5	24.7	98.8	1.9
	10	30.2	100.6	1.9
	20	40.1	100.2	2.1
Urine	0	0	Not detected	–
	10.0	9.4	94.0	1.9
	15.0	15.1	100.6	2.3
	30.0	31.1	103.4	1.7
Plasma	0	0	Not detected	–
	25.0	26.1	104.4	1.5
	35.0	34.4	98.3	2.1
	50.0	50.0	100.1	2.1

## Conclusions

The electrochemical behavior of ACV was investigated on an electrochemical sensor modified with magnetic CdO nanoparticles. The electrochemical response was a diffusion control process. High sensitivity and low detection limit (300 nM), as well as easy preparation and easy surface recovery of the modified electrode, are the main advantages of this sensor. The proposed sensor as an effective electrochemical sensor was used to the ACV determination by DPV technique in biological fluids.

## Data Availability Statement

The raw data supporting the conclusions of this article will be made available by the authors, without undue reservation.

## Ethics Statement

The studies involving human participants were reviewed and approved by ethics committee of Iran University of Medical Sciences. Written informed consent from the participants was not required to participate in this study in accordance with the national legislation and the institutional requirements.

## Author Contributions

All authors listed have made a substantial, direct and intellectual contribution to the work, and approved it for publication.

## Conflict of Interest

The authors declare that the research was conducted in the absence of any commercial or financial relationships that could be construed as a potential conflict of interest.
